# Flower fertilization optimization algorithm with application to adaptive controllers

**DOI:** 10.1038/s41598-025-89840-1

**Published:** 2025-02-21

**Authors:** Hazim Albedran, Shaymaa Alsamia, Edina Koch

**Affiliations:** 1 Mechanical Engineering Department, Faculty of Engineering, University of Kufa, Kufa, Iraq; 2 Department of Structural and Geotechnical Engineering, Széchenyi István University, Hungary University, Gyor, Hungary

**Keywords:** PID controller, Engineering control, Optimization, Metaheuristics, Computational intelligence, Mechanical engineering, Computer science, Software

## Abstract

This article presents the Flower Fertilization Optimization Algorithm (FFO), a novel bio-inspired optimization technique inspired by the natural fertilization process of flowering plants. The FFO emulates the behavior of pollen grains navigating through the search space to fertilize ovules, effectively balancing exploration and exploitation mechanisms. The developed FFO is theoretically introduced through the article and rigorously evaluated on a diverse set of 32 benchmark optimization problems, encompassing unimodal, multimodal, and fixed-dimension functions. The algorithm consistently outperformed 14 state-of-the-art metaheuristic algorithms, demonstrating superior accuracy, convergence speed, and robustness across all test cases. Also, exploitation, exploration, and parameter sensitivity analyses were performed to have a comprehensive understanding of the new algorithm. Additionally, FFO was applied to optimize the parameters of a Proportional-Integral-Derivative (PID) controller for magnetic train positioning—a complex and nonlinear control challenge. The FFO efficiently fine-tuned the PID gains, enhancing system stability, precise positioning, and improved response times. The successful implementation underscores the algorithm’s versatility and effectiveness in handling real-world engineering problems. The positive outcomes from extensive benchmarking and practical application show the FFO’s potential as a powerful optimization tool. In applying multi-objective PID controller parameter optimization, FFO demonstrated superior performance with a sum of mean errors of 190.563, outperforming particle swarm optimization (250.075) and dynamic differential annealed optimization (219.629). These results indicate FFO’s ability to achieve precise and reliable PID tuning for control systems. Furthermore, FFO achieved competitive results on large-scale optimization problems, demonstrating its scalability and robustness.

## Introduction

While extensive research over the past few decades has resolved many linear control problems, most real-world systems—including ones that control vehicles, aircraft^1^, and robots^2^ are nonlinear. Nonlinear systems do not follow the straightforward laws of superposition and homogeneity that linear systems do. Instead, they exhibit varied behavior across different operating regions. This complexity makes designing effective controllers for nonlinear systems particularly challenging^3^. Despite advancements in nonlinear control methods like adaptive control, fuzzy control, and model predictive control MPC, the PID Proportional-Integral-Derivative PID controller is still widely used in industry. Approximately 90 percent of controllers are PID in the industry, underscoring the importance of simplicity and ease of use in engineering^4^. The key point for a successful PID controller lies in properly tuning its proportional, integral, and derivative gains. When applying a PID controller to a nonlinear system, many adjustments must be considered for nonlinear effects. On the other hand, optimization algorithms have increasingly been used to tune PID gains^3^ for many years. While traditional tuning methods like Ziegler–Nichols, Cohen-Coon, and heuristic strategies have been extensively researched and often yield satisfactory results for linear systems, they fall short when applied to nonlinear systems. Due to the challenges of applying PID controllers to nonlinear systems, hybrid approaches combine the simplicity of PID with the robustness of soft computing techniques to enhance system performance. Applying AI techniques to the gains tuning has proven to be more efficient than conventional methods. Researchers have employed various optimization algorithms to tune PID parameters over the past few decades^1^. ANNs, in particular, offer high computational speed based on previous system behaviors, while fuzzy logic controllers adjust PID gains based on error and change in error, though they require expert knowledge to define appropriate membership functions. Despite their widespread use, metaheuristic algorithms^5^often suffer from issues like parameter selection and computational speed. Consequently, hybrid methods combining ANN and Particle Swarm Optimization (PSO)^6^ are being explored^7^. PSO has demonstrated its efficiency in solving complex problems, and neural networks can be utilized for tuning PID gains. The usage of optimization algorithm in tuning PID parameters specifically cannot be restricted by analyzing the results obtained from PSO or GA^8^. Any algorithm has its own advantages and drawbacks, one algorithm can be superior on a problem and the same algorithm can be the worst choice for another problem. Researchers have been developing metaheuristic algorithms for many decades, inspiring their works from nature or human activity. Metaheuristics are used to find an unknown solution inside predictable search limits, and the solution usually takes the form of a maximum or minimum of a specific quantity. Thus, one of the limitations of these algorithms is that the search space should be predefined. In many cases, defining search limits is a real problem and is chosen by trial and error or according to the designer’s ingenuity. However, finding the best possible solution in a limited space is not easy, especially when we know that there are infinite solutions for any continuous search space. However, this is not the case for combinatorial optimization with discrete search space where there are a finite number of solutions. The continuous inspiration of new algorithms is always required because there is no super algorithm for all the problems and applications^9^. The statistical results of the new and old algorithms enable the final user to decide which algorithm is appropriate for a specific optimization problem.Some of the physical and human-made phenomena have optimization procedures that have been implemented successfully to develop efficient optimization algorithms. It is not necessary to follow the same procedure in real life. Still, at least the inspiration base should give an abstract idea that can be modeled mathematically to an optimizer. For example, DDAO: Dynamic differential annealed optimization^10^ was inspired by dual-phase steel manufacturing. SCA: Sine Cosine Algorithm^11^ was inspired by sine and cosine functions. MVO: Multi-Verse optimizer^12^ by cosmological concepts: white, black, and wormholes. Natural inspired algorithms are another set of optimization algorithms that natural individuals have inspired in real life^13^ where there is no shared knowledge among these individuals. The rational processes that are employed by some biological species provide a predefined logic for the developers to imitate these species and develop new optimizers. For example, GWO: Grey wolf optimizer^14^ was inspired by hunting mechanism of the grey wolf. MFO: Moth-flame optimization algorithm^15^ by navigation of moths in nature. SMA: Slime Mould Algorithm^16^ inspired by the behavior the of slime mold in nature. Swarm optimization algorithms use the collective intelligence of a decentralized and self-organized population of individuals to find the global optimum. Collective intelligence can only be reached by homogenous individuals that share their knowledge among themselves and cannot be reached by a single agent in the population^17^. This definition expresses the difference between swarm optimization algorithms and naturally inspired algorithms. The following are some of the well-known swarm optimizers. Salp Swarm Algorithm^18^ inspired by salp swarm, Harris Hawks Optimization^19^ by the behavior of Harris’ hawks during chasing, adaptive exploration artificial bee colony AEABC by honey bee colony^20^. The evolutionary computations can be divided into four main groups: genetic algorithms, evolutionary strategies, evolutionary programming, and genetic programming. Evolutionary computations have been focusing on the use of natural evolution mechanisms to improve numerical values representing a set of points in a space. These points or candidate solutions are associated with a given application for finding an optimal solution by utilizing the concept of “survival of the fittest”. The way of applying some operators on the algorithm process and the representation form of the individuals specify the possible variations on the algorithm, and the following are examples: GA: Genetic algorithm^21^ inspired by evolutionary theory, F3EA^22^ by Find-Fix-Finish-Exploit-Analyze targeting process.For criteria of selection an algorithm, it is recommended to see topic-related review papers which are written for specific problems like academic scheduling problems^23^, oil and gas industry^24,25^, and interactive clustering^26^. Other works have reviewed specific optimization algorithms to reveal the reliability of these algorithms on different problems and fields^27–29^. In brief, the validity and usability of an optimization algorithm can only be conducted by testing that algorithm on a specific benchmark that represents a mathematical problem or an engineering application. This is the core of the no-free lunch theorem^9^. In this article, we present the flower fertilization optimization (FFO) algorithm, a novel optimization method inspired by the natural fertilization processes of flowering plants. We extensively evaluate the FFO algorithm by employing it on a total of 32 benchmark test functions. The FFO algorithm was compared with 14 optimization algorithms on these benchmarks. Remarkably, the FFO algorithm demonstrated competitive performance against all 14 competitors and, in several cases, achieved superior results. The successful optimization of such a diverse set of benchmarks comes from the reliability and effectiveness of the FFO for a wide range of optimization problems. Due to these promising results, the FFO algorithm was employed to develop an optimized artificial neural network-based controller; an adaptive PID controller for a magnetic train system. The optimization process was executed multiple times with random initial conditions and reference positions for the magnetic train. The resulting optimal PID gain values and their corresponding performance metrics, which are overshoot, settling time, steady-state error, and control effort, were collected to form a comprehensive dataset. This dataset was then used to train an artificial neural network model capable of predicting optimal PID gains for new initial conditions in real time. The combination of the FFO algorithm for dataset generation and the ANN for real-time prediction provides a robust and adaptive PID control solution capable of handling varying initial conditions and reference positions in the magnetic train system.

The contributions of this article as follows:

1. Development of a Novel Optimization Algorithm: Flower Fertilization Optimization FFO
2. Application FFO to PID Controller Optimization
3. Development of an optimized ANN-Based Real-Time PID Controller

In recent years, the metaheuristic community has faced increasing criticism regarding the proliferation of metaphor-based algorithms, as noted in studies such as^30^ and^31^. This indicates issues such as structural bias, lack of genuine novelty, and misleading experimental validations in many widely cited algorithms, including the grey wolf optimizer (GWO)^32^, whale optimization algorithm (WOA)^33^, and chimp optimization algorithm (ChOA).^34^ Despite their popularity, these algorithms often lack robust foundations and are prone to overfitting specific benchmark problems, as discussed in^35,36^, and^37^. By grounding its design in well-established optimization principles, FFO aims to provide a robust and transparent tool that avoids the pitfalls commonly associated with metaphor-based algorithms. The primary objective of this work is to introduce and validate the new FFO algorithm rather than criticizing existing metaphor-based algorithms. However, algorithms such as GWO and WOA are included in this study as widely recognized optimization tools, regardless of their theoretical basis, to serve as benchmarks for evaluating the performance of the proposed FFO algorithm. The structure of this paper is as follows: Sect. “Related works” shows the related works. Section “Inspiration background” provides the inspiration background for the flower fertilization optimization algorithm, detailing its biological foundation and mathematical modeling. Section “Experiments” outlines the experiments conducted to evaluate the performance of FFO on various benchmark functions and its comparisons with other metaheuristic algorithms. Section “The time complexity” has explored the time complexity of the FFO algorithm, demonstrating its computational efficiency across different problem sizes. Section “Wilcoxon signed-rank test analysis” presents the wilcoxon signed-rank test analysis to statistically validate the performance differences between FFO and competing algorithms. Section “Exploitation analysis” analyzes the exploitation capabilities of the FFO algorithm, showcasing its strength in refining solutions within promising regions of the search space. Section “Exploration analysis” examines the exploration capabilities of the FFO algorithm, revealing its ability to diversify the search and avoid local optima. Section “Sensitivity of the FFO algorithm” investigates the sensitivity of the FFO algorithm to key parameters and evaluates the impact of design choices on its performance. Section “Optimized PID controller for magnetic train positioning” demonstrates the application of FFO in optimizing PID controllers for magnetic train positioning, illustrating its practical engineering utility. Section “Conclusion” concludes the paper by summarizing the key findings and contributions, along with recommendations for future research directions.

## Related works

Over the years, new metaheuristic algorithms like the Draco Lizard Optimizer (DLO)^38^ and the Eurasian Lynx Optimizer (ELO)^39^ have raised the bar for balancing exploration and exploitation. Inspired by gliding Draco lizards, DLO^30^ stands out for its efficiency and global search strength. ELO, which models the hunting style of Eurasian lynxes, uses adaptive techniques—like Lévy flight and Gaussian mutation—to achieve top-notch results across a range of test functions and real-world challenges. Together, these advancements show how bio-inspired methods continue to evolve and take on more complex engineering problems. Recent work has built on these breakthroughs, using improved algorithms in areas such as load frequency control, renewable energy systems, and power electronics. These newer approaches deliver more stable systems, faster transient response, and lower steady-state errors. For instance, the RUN optimizer combined with pattern search has been used to design robust controllers for automatic voltage regulators, leading to notable decreases in rise and settling times^40^. Meanwhile, combining atom search optimization with particle swarm optimization has shown superior dynamic performance for controlling higher-order systems^41^. Fractional-order controllers optimized with the Pelican Optimization Algorithm significantly improve both steady-state behavior and stability in DC-DC converters^42^. Other studies use multi-stage controllers fine-tuned via the GEO algorithm, effectively managing DC-DC buck converter performance under different operating scenarios^43^. In load frequency control, a simulated annealing-based quadratic interpolation optimizer successfully stabilizes multi-area systems, even as loads fluctuate^44^. Moreover, the hunger games optimizer paired with pattern search has proven extremely robust and reliable in handling various engineering tasks^45^. Hybrid optimization has also grown in popularity. For instance, BDGOA has been applied to PV-thermal hybrid systems to tackle the nonlinearities and uncertainties inherent in renewable energy^46^. In wind energy, the reptile search algorithm excels at tuning PID controllers for doubly fed induction generators, outperforming methods like PSO, GSA, and BFO in terms of transient response and reliability^47^. Collectively, these strides underscore how bio-inspired optimization is driving real-world solutions for complex control problems—turning theoretical advances into practical benefits. It is suitable to mention that inspiration new metaheuristic algorithms is a continues work as seen in the next examples. The potter optimization algorithm^48^ is a metaheuristic inspired by the pottery-making process, balancing exploration (broad changes) and exploitation (precise refinements). The carpet weaving optimization algorithm^49^is a human-inspired metaheuristic based on the interaction between a carpet weaver and a map reader. The fossa optimization algorithm^50^is a bio-inspired metaheuristic based on the fossa’s two-stage hunting strategy, balancing exploration (initial attack) and exploitation (chase). The addax optimization algorithm^51^is a nature-inspired metaheuristic based on the addax’s foraging and digging behaviors. The dollmaker optimization algorithm^52^ is a human-inspired metaheuristic based on the doll-making process, balancing exploration (large material changes) and exploitation (fine adjustments). The sculptor optimization algorithm^53^ is a metaheuristic inspired by the sculpting process, balancing exploration (extensive material changes) and exploitation (fine details). The sales training based optimization algorithm^54^ is a metaheuristic inspired by human behaviors in sales training. The orangutan optimization^55^ is a metaheuristic inspired by orangutan foraging and nesting behaviors. The tailor optimization^56^is a human-inspired metaheuristic based on tailoring, balancing exploration (fabric adjustments) and exploitation (fine garment details). The spider-tailed horned viper optimization^54^ is a nature-inspired metaheuristic based on the viper’s hunting strategy, balancing exploration (searching terrain) and exploitation (targeting prey).

## Inspiration background

The FFO algorithm is a novel biological optimization algorithm inspired by the natural process of flower fertilization in plants. This algorithm models the behavior of pollen grains as they search for and fertilize ovules (eggs), drawing parallels between biological phenomena and optimization techniques. This section formulates and describes the FFO algorithm in detail.

### Iterative fertilization process

In nature, pollen grains fertilize ovules during one or several pollination events. This is analogous to iterative optimization processes, where we seek the optimal solution over multiple iterations. Each iteration represents a new attempt at finding a better solution, similar to how multiple pollination events increase the chances of successful fertilization.

### Probability of successful fertilization and population size

The probability *P* of successful fertilization is proportional to the amount of pollen released:

1 $$P\propto\; Amount \;of\; Pollen$$

This corresponds to the population size in optimization algorithms. Increasing the number of solutions (pollen grains) enhances the probability of finding the optimal solution. This concept aligns with population-based algorithms, where a larger population increases the chances of exploring the search space effectively and discovering the best solution.

### Decreasing velocity of pollen grains

Pollen grains lose velocity over time due to environmental factors, and those with zero velocity are considered non-viable (dead). To model this behavior, we introduce a velocity reduction coefficient γ, which is damped over iterations using a damping factor β (where 0 < β < 1)

2 $${\gamma }^{t+1}=\beta {\upgamma }^{t}$$

This reduction simulates the decreasing mobility of pollen grains over time, mirroring how solutions in an optimization algorithm might slow their exploration to focus on promising areas.

### Pollen grains and search space

In the FFO, pollen grains represent potential solutions, and the physical space in which they move corresponds to the search space in optimization. The movement and interaction of pollen grains within this space model how solutions explore and exploit the search landscape to find the optimal point.

### Survival and mixing of pollen populations

Some pollen grains remain viable longer than others. In the algorithm, pollen from previous iterations is combined with pollen from the current iteration. They are mixed, and only the best pollen grains (solutions) survive for a fixed number of trials *N*. This reflects the concept of elitism in optimization, where the best solutions are preserved and carried forward to improve convergence toward the optimum.

### Objective function and proximity to the ovule

The objective function in optimization corresponds to how close a pollen grain is to the ovule. In minimization problems, the best solution has the lowest objective function value, while in maximization problems, it has the highest value. This parallels the goal of pollen grains to reach and fertilize the ovule, where proximity increases the likelihood of successful fertilization.

### Initial positions, velocities, and search strategies

Initially, all solutions (pollen grains) have random positions and velocities, spreading out in the search landscape. The global search mechanism uses Lévy flights to model the long-distance movement of pollen grains:

3 $$X_{i}^{t + 1} = L\left( {X_{i}^{t} - V_{i}^{t} } \right)\;({\text{i}}\, = \,{1},{2} \ldots ,{\text{ N}}),$$

where ${X}_{i}^{t}$ is a solution while ${V}_{i}^{t}$ represents its velocity, the superscript t refers to the previous status of the solution while *t* + 1 refers to the status of the updated solution, *L* Step size drawn from a Lévy distribution, and *i* is the index of the solution in the population of number *N*. Lévy flights enable the algorithm to explore the search space globally by allowing large jumps, preventing premature convergence to local optima.

The formula for $L$ in the Levy function is derived from the Lévy flight distribution. It is typically expressed as:

4 $$L=\frac{u}{{\left|v\right|}^{1/\xi }}$$

where $u$ and $v$ are random variables drawn from normal distributions:

5 $$u\sim N(0,\sigma 2)$$

6 $$v\sim N(0,1)$$

$$\sigma$$

is calculated as

7 $$\sigma ={\left(\frac{\Gamma \left(1+\xi \right).sin\left(\pi \xi /2\right)}{\Gamma \left(\frac{\left(1+\xi \right)}{2}\right)\xi . {2}^{\frac{\xi -1}{2}}}\right)}^{1/\xi }$$

where $\Gamma$ is the Gamma function.$\xi$ is the Lévy distribution parameter, typically set between 1 and 2 (commonly $\xi =1.5)$ .

### Local search via velocity reduction

As time progresses, the velocity of pollen grains decreases, focusing the search locally. This is modeled using an exponential decay function:

8 $${V}_{i}^{t+1}={V}_{i}^{t}{e}^{\left(\frac{-1}{{\gamma }^{t+1}}\right)}$$

where ${\gamma }^{t+1}$ is a reduction coefficient that has to be damped over iterations, ${V}_{i}^{t+1}$ and ${V}_{i}^{t}$ are the velocity of the solution in the current iteration and previous iteration, respectively. A damping factor β (e.g., β = 0.95) ensures the velocity decreases over iterations. The initial value of $\gamma$ can be taken 1 and reduced over iterations.

### Movement toward the best solution

Imagining the population of solutions moving as a cohesive group toward the optimal solution, there is one solution at the very front end ${X}_{first}^{t}$, one at the very back end ${X}_{end}^{t}$, and there is one at the middle of the block ${X}_{middle}^{t}$. The values of the solutions change, and the following equation is to express this change in position:

9 $${X}_{i}^{t+1}=\frac{{X}_{first}^{t}+{X}_{end}^{t}{+X}_{middle}^{t}}{3}\delta$$

where $\delta$ is a random mutation term for the *i*-th solution, $\delta \in [\text{0,1}]$, introducing diversity. This equation encourages solutions to move toward the best-performing areas while maintaining diversity through random perturbations. Equation (5) is calculated by sorting the population of the solutions and easily we can find ${X}_{first}^{t}$ , ${X}_{end}^{t}$, and ${X}_{middle}^{t}$.

### Mathematical model of the FFO

By combining the global and local search mechanisms, the update rule for each solution becomes:

10 $${X}_{i j}^{t+1}={X}_{i j}^{t}-{V}_{i j}^{t}{e}^{\left(\frac{-1}{{\gamma }^{t+1}}\right)}+L\left({X}_{i}^{t}-{V}_{i j}^{t}\right)-\frac{{X}_{first}^{t}+{X}_{end}^{t}{+X}_{middle}^{t}}{3}\delta$$

where *j* is an index of the component in a solution of length *m*.

Thus, the algorithm pseudocode is:

Inputs:

- f(x): Objective function to optimize
- var: Number of decision variables
- minL, maxL: Lower and upper search space limits
- Agents: Population size
- Iteration: Maximum number of iterations
- $\upgamma$: Velocity reduction coefficient (initial value)
- β: Damping factor for velocity reduction

Initialization:

1. Create an empty structure pollen with attributes:
  Position: solution in the search space.
  Velocity: velocity vector.
  Cost: cost value based on f(x).

2. Initialize population of agents pollen grains:
  • Randomize position within bounds [minL, maxL].
  • Set initial velocity equal to position.
  • Compute cost using f(x).

3. Initialize an array BestCosts to store the best cost at each iteration.

Main Loop:

For *t* from 1 to Maximum number of iterations:

1. Initialize a new pollen population (newpollen).
2. For each pollen grain in the current population:
  Calculate $$K=\frac{{X}_{Best}^{t}+{X}_{Median}^{t}{+X}_{Worst}^{t}}{3}$$
  Generate a Lévy step size L
  Compute $$\Delta S=L\left({X}_{current}-{V}_{current}\right)$$
  Update velocity using:${V}_{new}={V}_{old}. {e}^{\left(\frac{-1}{{\gamma }^{t+1}}\right)}$
  Update position using: ${X}_{new}={X}_{current}-{V}_{new}+\Delta S-K.rand()$
  Clamp ${X}_{new}$ to [minL, maxL] to ensure it stays within bounds.
  Compute the cost of the ${X}_{new}.$
  Add the ${X}_{new}$ to newpollen.

3. Merge and Sort Populations:
  Combine the current population with newpollen.
  Sort the population based on Cost in ascending order.

4. Competitive Exclusion:
  Retain the top agents pollen grains.

5. Update Best Solution:
  Store the best pollen grain and its cost.
  Record the best cost in BestCosts[it].

6. Reduce Velocity Coefficient:
  Update $${\upgamma }_{new}={\upgamma }_{old}.\upbeta$$

**Fig. 1:**
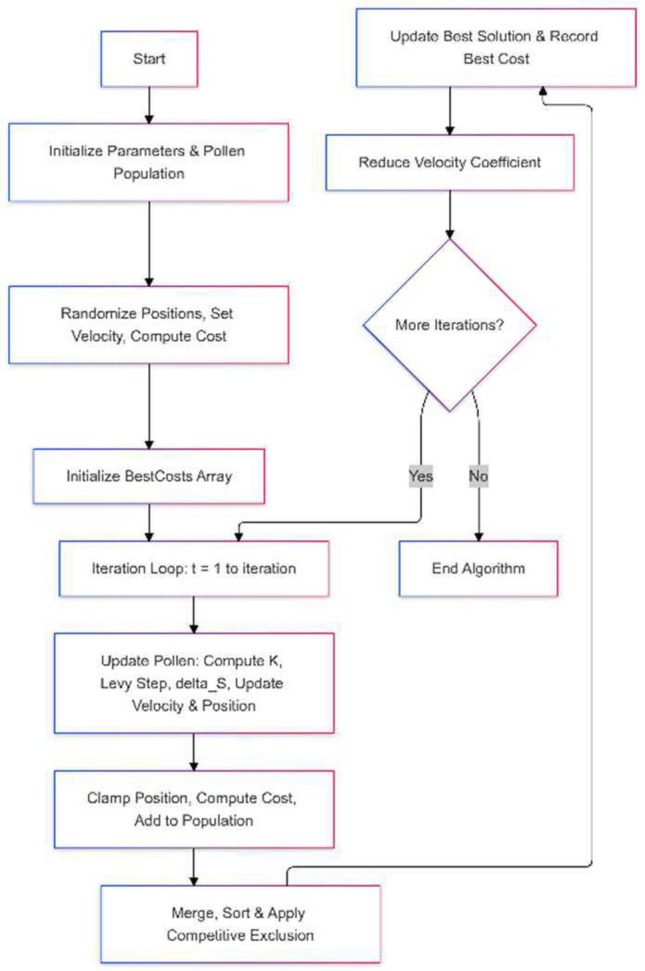
The FFO algorithm flowchart.

Figure 1 shows the flowchart of the FFO algorithm while, the source code in Python can be downloaded at: https://drive.google.com/file/d/1HcgO7n6KFn5yLUZWz_g5GvQ6OY9ivMkd/view?usp=sharing

The Flower Fertilization Optimization Algorithm effectively balances exploration and exploitation by modeling the natural behaviors of pollen grains. Lévy flights allow solutions to explore the search space globally. Velocity reduction and movement toward the best solutions focus the search locally. Random mutations and mixing of populations prevent premature convergence. This biologically inspired approach utilizes natural processes to enhance optimization performance, making it suitable for solving complex problems in various domains.

## Experiments

The FFO algorithm was examined by optimizing 48 benchmark functions and compared with 14 optimization algorithms in different run conditions. The experiments are accomplished based on the maximum iteration number as well as the maximum elapsed time. Some statistical results are adopted from other articles, and the FFO algorithm is run according to the run conditions of those articles to have fair comparisons. Table 1 shows a summary table detailing the control parameter values of the optimization algorithms used in the manuscript.

**Table 1:** Control parameter values of the optimization algorithms.

Algorithm	Key parameters
Particle swarm optimization	Max velocity): 6
	Inertia weight : [0.9, 0.2]
	Cognitive coefficient : 2
	Social coefficient : 2
Sine cosine algorithm	$a$ decreases linearly from 2 to 0 during iterations
	Random coefficients: r_1_,r_2_,r_3_,r_4_
Whale optimization algorithm	Coefficients: $a$ (linearly decreases from 2 to 0), A,C (random coefficients)
	Adaptive mechanism based on $p$: [0, 1]
Ant lion optimizer	Roulette Wheel selection mechanism
	Random walk for ants and antlions
Butterfly optimization algorithm	Sensory modality (*c*): 0.01
	Power exponent ($a$): 0.1
	Switch probability (*p*): 0.8
Harris hawks optimization	Escaping energy: E (adaptive)
	Exploration and exploitation mechanisms based on random coefficients
Grey wolf optimizer	Hierarchical roles: Alpha, Beta, Delta
	Adaptive coefficients (a,A,C): Linearly decreasing and random
Dynamic differential annealing optimization	Max sub-iterations: 100
	Initial temperature (T_0_): 2000
	Cooling rate (α): 0.95
Flower fertilization optimization	Velocity reduction coefficient (x): 1
	Damping factor (d): 0.95
	Combination of Lévy flights and local search

### Time-based mathematical optimization

This section presents the results of optimizing 23 mathematical optimization functions. These test functions are detailed and employed in,^14^ with a general description shown in Table 2.

**Table 2:** Unimodal, multimodal, and fixed-dimensions benchmarks.

Prob	Type	D	Global
F1-F7	Unimodal	30	0
F8	Multimodal	30	418.9829 $\times$ 5
F9-F13		30	0
F14		30	− 4.687
F15-F16		30	− 1
F17	Fixed-dimension	2	0.398
F18		2	3
F19		3	− 3.86
F20		6	− 3.32
F21		4	− 10.1532
F22		4	− 10.4028
F23		4	− 10.5363

In this experiment, the FO algorithm was compared with the six metaheuristics^10^ shown in Table 3, 4, and 5. In this test, each algorithm has a maximum time one second with 25 population size to return results over 51 independent runs. In this experiment, the elapsed time is the stop criteria for the competitive algorithms, we have set iteration number 100 for ant lion optimizer ALO^57^, harris hawks optimization HHO^19^, butterfly optimization algorithm BOA^58^, grey wolf optimizer GWO^32,59^, whale optimization algorithm WOA^33,60^ and sine cosine algorithm SCA^11,61^ even when other algorithms; PSO, DDAO, and FFO don’t use this parameter. Finally, all algorithms have to exhaust the one-second to stop. Axiomatically, each algorithm in this test will spend a different number of function evaluations due to their differences in complexity and structure. The MATLAB codes of the algorithms used in this experiment can be downloaded from: https://www.mathworks.com/matlabcentral/fileexchange/ .

**Table 3:** Results on unimodal benchmarks.

Algorithm		ALO	HHO	BOA	GWO	PSO	SCA	WOA	DDAO	FFO
F1	Best	2.714E + 03	4.019E−70	1.151E−24	2.865E−10	2.602E−01	1.952E + 01	1.099E−45	3.179E−04	**1.570E**−**175**
	Mean	6.735E + 03	4.544E−46	2.466E−22	3.812E−09	1.263E + 00	1.319E + 03	1.282E−32	4.255E + 00	**1.101E**−**78**
	STD	2.473E + 03	3.183E−45	1.324E−21	5.298E−09	1.009E + 00	1.445E + 03	8.985E−32	7.206E + 00	**7.784E**−**78**
F2	Best	2.106E + 01	2.561E−34	**2.186E**−**137**	6.003E−07	1.004E + 00	8.368E−02	2.242E−31	3.831E−03	2.725E−86
	Mean	8.453E + 01	5.771E−26	1.068E−01	2.782E−06	2.685E + 00	1.487E + 00	2.426E−24	7.725E−01	**6.209E**−**32**
	STD	4.125E + 01	2.542E-25	7.548E-01	1.573E-06	1.054E + 00	1.120E + 00	1.279E-23	7.813E-01	**4.390E-31**
F3	Best	6.228E + 03	1.293E−51	9.467E−16	1.268E + 00	3.258E + 02	7.588E + 03	1.612E + 04	3.988E−03	**4.091E**−**122**
	Mean	2.033E + 04	4.483E−31	8.160E−11	3.369E + 01	9.179E + 02	2.233E + 04	3.017E + 04	2.336E + 01	**2.812E**−**26**
	STD	9.802E + 03	**2.266E**−**30**	4.971E−10	3.507E + 01	3.007E + 02	9.471E + 03	5.509E + 03	3.675E + 01	1.988E−25
F4	Best	2.026E + 01	1.050E−38	9.280E−22	1.751E−02	2.687E + 00	3.116E + 01	1.618E + 00	3.345E−03	**2.440E**−**85**
	Mean	3.163E + 01	9.604E−26	1.404E−18	1.498E + 00	5.076E + 00	5.496E + 01	5.897E + 01	5.137E−01	**3.748E**−**36**
	STD	6.434E + 00	6.105E−25	6.793E−18	9.678E−01	1.708E + 00	1.149E + 01	2.439E + 01	9.576E−01	**2.650E**−**35**
F5	Best	2.051E + 05	**8.734E**−**07**	2.873E + 01	2.727E + 01	2.442E + 02	1.880E + 04	2.344E−05	2.901E + 01	2.866E + 01
	Mean	2.660E + 06	**1.191E**−**04**	2.882E + 01	2.864E + 01	7.168E + 02	3.913E + 06	2.114E + 01	1.009E + 02	2.890E + 01
	STD	1.988E + 06	**2.113E**−**04**	3.766E−02	1.179E + 00	5.497E + 02	6.465E + 06	1.044E + 01	3.434E + 02	8.666E−02
F6	Best	1.984E + 03	**4.797E**−**09**	1.954E + 00	1.818E + 00	1.999E−01	4.735E + 01	4.923E−12	6.847E + 00	4.879E + 00
	Mean	6.766E + 03	7.151E−06	3.623E + 00	3.298E + 00	1.351E + 00	1.039E + 03	**4.010E-08**	1.061E + 01	6.155E + 00
	STD	2.565E + 03	1.473E−05	7.946E−01	7.180E−01	7.730E−01	1.024E + 03	**2.503E**−**07**	8.517E + 00	5.472E−01
F7	Best	6.439E−01	7.030E−06	**5.945E**−**06**	3.264E−03	7.520E−01	7.222E−02	9.559E−05	5.650E−03	2.183E−05
	Mean	2.974E + 00	**2.031E**−**04**	2.839E−04	1.489E−02	3.428E + 00	2.417E + 00	9.514E−03	7.262E−02	3.058E−04
	STD	1.827E + 00	**1.727E**−**04**	3.570E−04	6.836E−03	3.352E + 00	2.785E + 00	1.224E−02	5.211E−02	2.259E−04

Significant values are in [bold]

**Table 4:** Results on multimodal benchmarks.

Algorithm		ALO	HHO	BOA	GWO	PSO	SCA	WOA	DDAO	FFO
F8	Best	− 7.075E + 03	− **1.257E + 04**	− 3.355E + 03	− 8.092E + 03	− 6.227E + 03	− 4.930E + 03	− **1.257E + 04**	− 4.894E + 03	− 5.293E + 03
	Mean	− 5.429E + 03	− **1.257E + 04**	− 2.088E + 03	− 5.842E + 03	− 3.695E + 03	− 4.216E + 03	− 1.242E + 04	− 4.190E + 03	− 4.160E + 03
	STD	5.678E + 02	**5.764E−03**	4.771E + 02	8.669E + 02	7.827E + 02	2.461E + 02	3.594E + 02	2.817E + 02	5.294E + 02
F9	Best	6.407E + 01	**0.000E + 00**	**0.000E + 00**	1.060E + 01	6.035E + 01	1.004E + 01	0.000E + 00	5.204E**−**03	**0.000E + 00**
	Mean	1.119E + 02	**0.000E + 00**	**0.000E + 00**	3.275E + 01	1.199E + 02	1.151E + 02	1.895E**−**14	8.720E + 00	**0.000E + 00**
	STD	2.476E + 01	**0.000E + 00**	**0.000E + 00**	1.439E + 01	3.997E + 01	5.020E + 01	7.523E**−**14	3.352E + 01	**0.000E + 00**
F10	Best	9.450E + 00	**8.882E−16**	**8.882E-16**	2.922E**−**06	1.193E + 00	2.156E + 00	**8.882E−16**	5.278E**−**03	**8.882E−16**
	Mean	1.503E + 01	**8.882E−16**	6.017E**−**14	1.139E**−**05	2.347E + 00	1.422E + 01	6.670E**−**15	9.702E**−**01	2.908E**−**15
	STD	1.352E + 00	**0.000E + 00**	4.112E**−**13	6.461E**−**06	4.357E**−**01	6.115E + 00	5.885E**−**15	1.046E + 00	1.760E**−**15
F11	Best	3.808E + 01	**0.000E + 00**	**0.000E + 00**	5.313E**−**10	2.082E + 00	1.093E + 00	0.000E + 00	1.103E**−**04	**0.000E + 00**
	Mean	6.596E + 01	**0.000E + 00**	**0.000E + 00**	4.441E**−**02	6.052E + 00	1.302E + 01	1.545E**−**03	8.581E**−**01	**0.000E + 00**
	STD	2.025E + 01	**0.000E + 00**	**0.000E + 00**	4.281E**−**02	3.174E + 00	1.279E + 01	6.595E**−**03	5.460E**−**01	**0.000E + 00**
F12	Best	2.196E + 01	**3.536E**−**08**	1.359E−01	9.962E−02	3.227E−03	2.245E + 02	2.672E−05	1.053E + 00	4.492E−01
	Mean	1.156E + 05	**2.380E**−**06**	3.241E−01	5.300E−01	7.441E−01	7.288E + 06	1.793E−04	1.732E + 00	8.711E−01
	STD	3.554E + 05	**2.453E**−**06**	1.016E−01	4.147E−01	8.905E−01	1.654E + 07	2.251E−04	5.695E−01	2.017E−01
F13	Best	1.635E + 04	**2.314E**−**07**	1.232E + 00	1.332E + 00	1.480E−01	5.889E + 04	2.016E−03	2.739E + 00	1.163E + 00
	Mean	3.038E + 06	**2.368E**−**05**	2.066E + 00	2.532E + 00	1.017E + 00	1.967E + 07	5.629E−02	5.248E + 00	2.952E + 00
	STD	3.620E + 06	**3.513E**−**05**	4.282E−01	5.053E−01	9.811E−01	2.294E + 07	6.304E−02	3.069E + 00	2.540E−01
F14	Best	9.980E−01	**9.980E**−**01**	**9.980E**−**01**	**9.980E**−**01**	**9.980E**−**01**	**9.980E**−**01**	**9.980E**−**01**	**9.980E**−**01**	**9.980E**−**01**
	Mean	7.151E + 00	**1.154E + 00**	1.232E + 00	2.285E + 00	4.122E + 00	2.275E + 00	1.984E + 00	1.947E + 00	5.289E + 00
	STD	4.325E + 00	**3.615E**−**01**	5.235E−01	1.597E + 00	2.707E + 00	1.945E + 00	2.362E + 00	1.167E + 00	3.049E + 00
F15	Best	7.022E−04	3.083E−04	3.099E−04	4.211E−04	4.081E−04	5.352E−04	3.075E−04	9.172E−04	**3.082E**−**04**
	Mean	6.387E−03	**3.977E**−**04**	4.121E−04	9.692E−04	9.461E−04	1.030E−03	4.968E−04	2.268E−03	1.024E−03
	STD	6.072E−03	2.917E−04	6.981E−05	5.294E−04	**1.745E**−**04**	2.965E−04	2.197E−04	9.713E−04	1.639E−03
F16	Best	−**1.032E + 00**	−**1.032E + 00**	−9.963E-01	−**1.032E + 00**	−**1.032E + 00**	−**1.032E + 00**	−**1.032E + 00**	−1.031E + 00	−**1.032E + 00**
	Mean	−1.016E + 00	−**1.032E + 00**	−1.333E-01	−1.032E + 00	−1.032E + 00	−1.031E + 00	−1.032E + 00	−1.028E + 00	−**1.032E + 00**
	STD	1.132E−01	3.170E−07	3.640E−01	1.854E−07	**3.921E**−**16**	1.879E−04	4.506E−16	4.503E−03	4.966E−03

Significant values are in [bold]

**Table 5:** Results on Fixed-dimensions benchmarks.

Algorithm		ALO	HHO	BOA	GWO	PSO	SCA	WOA	DDAO	FFO
F17	Best	**3.979E−01**	**3.979E−01**	0.000E + 00	**3.979E−01**	**3.979E−01**	3.980E**−**01	**3.979E−01**	**3.979E−01**	**3.979E−01**
	Mean	3.979E**−**01	3.979E**−**01	0.000E + 00	3.982E**−**01	3.979E**−**01	4.030E**−**01	3.979E**−**01	4.009E**−**01	3.984E**−**01
	STD	2.101E**−**11	1.080E**−**04	0.000E + 00	8.397E**−**04	3.886E**−**16	4.583E**−**03	2.500E**−**15	4.531E**−**03	1.558E**−**03
F18	Best	**3.000E + 00**	**3.000E + 00**	**3.000E + 00**	**3.000E + 00**	**3.000E + 00**	**3.000E + 00**	**3.000E + 00**	**3.001E + 00**	**3.000E + 00**
	Mean	4.059E + 00	**3.000E + 00**	3.002E + 00	3.001E + 00	**3.000E + 00**	3.001E + 00	**3.000E + 00**	3.070E + 00	5.289E + 00
	STD	5.241E + 00	1.862E**−**05	1.830E**−**03	4.540E**−**03	4.260E**−**15	1.629E**−**03	**2.197E−15**	7.001E**−**02	7.213E + 00
F19	Best	**−**3.863E + 00	**−**3.863E + 00	**−**3.846E + 00	**−**3.863E + 00	**−3.863E + 00**	**−**3.862E + 00	**−**3.863E + 00	**−**3.863E + 00	**−**3.862E + 00
	Mean	**−**3.853E + 00	**−**3.859E + 00	**−**3.165E + 00	**−**3.861E + 00	**−3.863E + 00**	**−**3.852E + 00	**−**3.862E + 00	**−**3.855E + 00	**−**3.816E + 00
	STD	2.509E**−**02	6.845E**−**03	3.829E**−**01	2.271E**−**03	**2.769E−15**	5.445E**−**03	2.119E**−**03	6.722E**−**03	1.183E**−**01
F20	Best	**−3.322E + 00**	**−**3.319E + 00	**−**2.616E + 00	**−3.322E + 00**	**−**3.322E + 00	**−**3.115E + 00	**−3.322E + 00**	**−**3.216E + 00	**−**3.300E + 00
	Mean	**−3.243E + 00**	**−**3.091E + 00	**−**1.350E + 00	**−**3.236E + 00	**−**3.278E + 00	**−**2.767E + 00	**−**3.245E + 00	**−**3.010E + 00	**−**2.796E + 00
	STD	9.683E**−**02	1.126E**−**01	4.436E**−**01	9.435E**−**02	**5.748E−02**	4.571E**−**01	2.118E**−**01	1.005E**−**01	3.108E**−**01
F21	Best	**−**1.015E + 01	**−**1.015E + 01	**−**9.516E + 00	**−**1.015E + 01	**−**1.015E + 01	**−**5.220E + 00	**−**1.015E + 01	**−**7.454E + 00	**−9.661E + 00**
	Mean	**−**5.367E + 00	**−**9.553E + 00	**−**7.479E + 00	**−**7.812E + 00	**−**5.723E + 00	**−**2.045E + 00	**−9.676E + 00**	**−**4.889E + 00	**−**4.154E + 00
	STD	2.721E + 00	1.642E + 00	1.212E + 00	3.230E + 00	3.027E + 00	1.370E + 00	1.918E + 00	**9.734E-01**	1.747E + 00
F22	Best	**−**1.040E + 01	**−**1.040E + 01	**−8.991E + 00**	**−**1.040E + 01	**−**1.040E + 01	**−**5.844E + 00	**−**1.040E + 01	**−**7.840E + 00	**−**1.008E + 01
	Mean	**−**5.489E + 00	**−**9.882E + 00	**−**6.191E + 00	**−**9.732E + 00	**−**6.330E + 00	**−**2.560E + 00	**−9.934E + 00**	**−**4.887E + 00	**−**4.577E + 00
	STD	3.183E + 00	1.580E + 00	**1.085E + 00**	2.040E + 00	3.365E + 00	1.386E + 00	1.879E + 00	1.029E + 00	1.762E + 00
F23	Best	**−1.054E + 01**	**−1.054E + 01**	**−**8.397E + 00	**−**1.053E + 01	**−1.054E + 01**	**−**6.678E + 00	**−1.054E + 01**	**−**9.678E + 00	**−**1.043E + 01
	Mean	**−**5.892E + 00	**−**9.475E + 00	**−**6.019E + 00	**−**9.920E + 00	**−**7.214E + 00	**−**2.997E + 00	**−1.036E + 01**	**−**4.771E + 00	**−**4.637E + 00
	STD	3.690E + 00	2.147E + 00	1.179E + 00	1.874E + 00	3.712E + 00	1.381E + 00	**1.228E + 00**	1.408E + 00	1.813E + 00

Significant values are in [bold]

The variability in standard deviation (STD) values across algorithms in Table 3 illustrates differences in their reliability and robustness. For example, FFO consistently achieves low STD values, indicating its robustness and ability to reliably converge to high-quality solutions. This is attributed to its well-balanced exploration and exploitation mechanisms. In contrast, algorithms like ALO and SCA exhibit higher STD values on certain functions, reflecting their sensitivity to initial conditions and a less reliable balance between exploration and exploitation. These results underscore the importance of designing algorithms capable of maintaining consistent performance across independent runs, particularly in applications where reliability is critical.

### Small scale optimization

The FFO algorithm was compared with seven optimization algorithms on five two-dimensional optimization problems, represented in Table 6. The experiment includes comparing the FFO algorithm with bat algorithm BA^62^, firefly algorithm FF^63^, multi-verse optimizer MVO^12,64^, Krill herd KH^65,66^, Squirrel search algorithm SSA^67^, spider monkey optimization algorithm SMO,^68^ and DDAO, of which their statistical results are known^67^. This test was achieved using 30 independent runs, a population size 50, and a maximum iteration number of 500. The results of the comparison are presented in Table 7.

**Table 6:** Small scale optimization problems.

Symbol	Function description	*D*	Range	*fmin*
F24	$f\left( x \right) = 0.5 + \frac{{\sin^{2} \left( {\sqrt {x_{1}^{2} + x_{2}^{2} } } \right) - 0.5}}{{\left( {1 + 0.001\left( {x_{1}^{2} + x_{2}^{2} } \right)} \right)^{2} }}$	2	[− 100, 100]	0
F25	$f\left( x \right) = x_{1}^{2} + 2x_{2}^{2} - 0.3\cos \left( {3\pi .x_{1} } \right) - 0.4\cos \left( {4\pi .x_{2} } \right) + 0.7$	2	[− 100, 100]	0
F26	$f\left( x \right) = 0.26\left( {x_{1}^{2} + x_{2}^{2} } \right) - 0.48x_{1} .x_{2}$	2	[− 10, 10]	0
F27	$f\left( x \right) = - \cos \left( {x_{1} } \right).\cos \left( {x_{2} } \right).\exp \left( { - \left( {x_{1} - \pi } \right)^{2} - \left( {x_{2} - \pi } \right)} \right)$	2	[− 100, 100]	− 1
F28	$f\left( x \right) = 4x_{1}^{2} - 2.1x_{1}^{4} + \frac{1}{3}x_{1}^{6} + x_{1} x_{2} + \left( { - 4 + 4x_{2}^{2} } \right)x_{2}^{2}$	2	[− 2, 2]	− 1.03163

**Table 7:** The FFO algorothm on small scale optimization.

Function		BA^67^	FF^67^	MVO^67^	KH^67^	SSA^67^	SMO^67^	DDAO^10^	FFO
F24	Best	9.715e−3	4.661e−3	2.151e−6	1.231e−7	0	1.997e−10	1.752e−9	**0**
	Mean	1.411e−1	1.18e−2	1.311e−3	3.360e−6	9.715e−4	3.934e−9	2.77e−6	**0**
	STD	1.069e−1	7.368e−3	3.352e−3	4.133e−6	2.964e−3	3.009e−9	1.622e−6	**0**
F25	Best	1.643	4.944e−8	1.002e−5	2.889e−8	**0**	2.133e−10	9.641e−7	**0**
	Mean	6.971e1	3.468e−6	3.595e−4	2.298e−7	**0**	4.24e−9	0.030674	**0**
	STD	1.119e2	2.663e−6	3.708e−4	2.945e−7	**0**	3.109e−9	0.01869	**0**
F26	Best	1.403e−12	2.967e−12	2.012e−10	2.168e−14	1.511e−29	3.540e−12	5.6686e−12	**1.099e**−**160**
	Mean	2.965e−11	3.139e−10	1.314e−8	8.869e−12	1.542e−25	3.146e−9	2.228e−6	**1.037e**−**155**
	STD	4.808e−11	2.930e−10	1.154e−8	1.285e−11	4.757e−25	3.125e−9	9.877e−7	**3.279e**−**155**
F27	Best	** − 1**	** − 1**	** − 1**	** − 1**	** − 1**	− **1**	− 0.9984	− 0.996998
	Mean	− 3.334e−2	− 7.333e−1	− 9.666e−1	− 9.666e−1	** − 1**	− 1	0.18036	− 8.322e−1
	STD	1.825e−1	4.497e−1	1.825e−1	1.825e−1	**0**	3.089e−9	− 0.8038	2.616e−1
F28	Best	** − 1.031**	** − 1.0316**	** − 1.0316**	** − 1.0316**	** − 1.0316**	− **1.0316**	− 1.0315	− **1.0316**
	Mean	− 0.95	** − 1.0316**	** − 1.0316**	** − 1.0316**	** − 1.0316**	− **1.0316**	0.00065	− 1.028
	STD	2.49e−1	3.944e−9	1.638e−7	6.358e−10	**4.516e-16**	3.21e−9	−1.03066	5.28e−3

Significant values are in [bold]

### Large scale optimization

The FFO algorithm compared with ALO, BOA, GWO, PSO, SCA, DDAO, bat algorithm BA,^62^ and tree-seed algorithm TSA^69^ on mathematical optimization problems shown in Table 8 plus F1. This is a challenging experiment where the algorithms have to return their best solutions over 51 independent runs, population size 25, maximum elapsed time 1 s, maximum number of iterations 100, and problem size 1000D. The comparison results are illustrated in Table 9, and remarkably, the FFO algorithm has almost overcome all of its competitors.

**Table 8:** Large-scale optimization problems.

Symbol	Function description	*D*	*Range*	*Fmin*
F29	$f\left( x \right) = 10n + \sum\limits_{i = 1}^{n} {\left[ {x_{i}^{2} - 10\cos \left( {2\pi \, x_{i} } \right)} \right]}$	1000	[− 5.12, 5.12]	0
F30	$f\left( x \right) = \sum\limits_{i = 1}^{n - 1} {\left[ {100\left( {x_{i + 1} - x_{i}^{2} } \right)^{2} + \left( {1 - x_{i} } \right)^{2} } \right]}$	1000	[− 2.048, 2.048]	0
F31	$f\left( x \right) = - a\exp \left( { - b\sqrt {\frac{1}{d}\sum\limits_{i = 1}^{d} {x_{i}^{2} } } } \right) - \exp \left( { - b\sqrt {\frac{1}{d}\sum\limits_{i = 1}^{d} {\cos \left( {cx_{i} } \right)} } } \right) + a + \exp \left( 1 \right)$	1000	[− 32.768, 32.768]	0
F32	$f\left( x \right) = \sum\limits_{i = 1}^{d} {\frac{{x_{i}^{2} }}{4000} - \prod\limits_{i = 1}^{d} {\cos \left( {\frac{{x_{i} }}{\sqrt i }} \right) + 1} }$	1000	[− 600, 600]	0

**Table 9:** FFO algorithm on large scale problems.

Function		F1	F29	F30	F31	F32
ALO	Best	1.119e6	1.246e4	9.614e4	1.946e1	9.814e3
	Mean	1.254e6	1.335e4	1.207e5	1.975e1	1.134e4
	STD	1.46e5	5.734e2	2.592e4	2.410e−1	1.421e3
BOA	Best	1.974e−15	0	**9.987e2**	1.295e−8	2.041e−6
	Mean	6.772e−15	3.352e−12	**9.988e2**	5.982e−8	5.576e−5
	STD	1.039e−14	1.596e−11	4.635e−2	9.753e−8	1.253e−4
GWO	Best	2.22e4	6.342e3	3.361e3	9.143	6.852e2
	Mean	3.115e4	6.904e3	4.88e3	1.012e1	9.727e2
	STD	9.322e3	2.694e2	8.969e2	9.856e−1	1.84e2
PSO	Best	9.59e4	1.477e4	4.21e5	1.700e1	8.141e2
	Mean	1.101e5	1.619e4	4.506e5	1.735e1	9.206e2
	STD	6.503e3	6.415e2	1.033e4	1.668e−1	4.401e1
SCA	Best	5.136e4	5.111e2	1.230e5	7.043	2.079e3
	Mean	8.271e5	1.743e3	2.323e5	1.66e1	8.356e3
	STD	3.4e5	9.181e2	3.852e4	3.292	2.524e3
DDAO	Best	1.354e−1	2.257e−1	9.989e2	2.438e−2	1.332
	Mean	6.267e2	5.742e2	1.038e3	3.310	8.483e1
	STD	2.029e3	1.083e3	8.985e1	2.154	1.576e2
BA	Best	9.12e5	1.266e4	5.406e4	1.936e1	1.024e4
	Mean	1.584e6	1.458e4	1.868e5	2.031e1	1.724e4
	STD	4.0e5	1.314e3	7.789e4	4.77e−1	4.975e3
TSA	Best	5.573e3	6.365e3	1.201e4	6.412	3.136e2
	Mean	1.501e4	1.021e4	2.794e4	9.04	1.087e3
	STD	1.273e4	2.161e3	9.291e3	1.595	5.194e2
FFO	Best	**7.966e**−**25**	**0**	9.989e2	**8.961e**−**13**	**0**
	Mean	**3.606e**−**18**	**0**	9.989e2	**5.036e**−**9**	**1.425e**−**8**
	STD	**2.574e**−**17**	**0**	**2.289e**−**2**	**3.497e**−**8**	**1.012e**−**7**

Significant values are in [bold]

## The time complexity

**Fig. 2:**
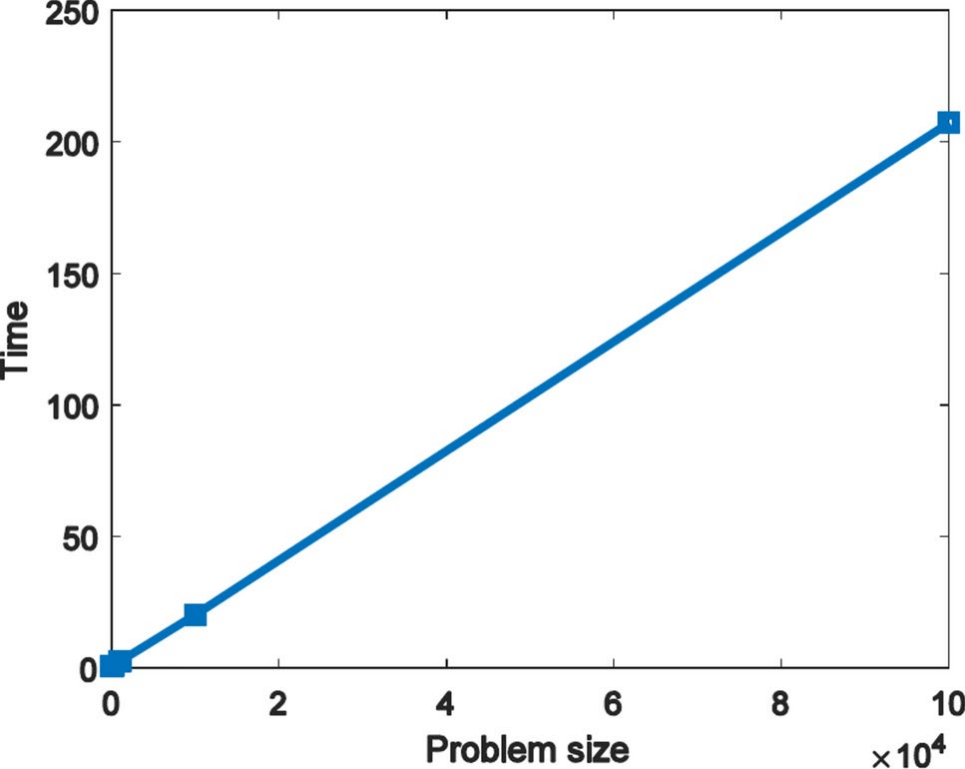
Runtime vs. problem size.

F1 was used to examine the complexity of the FFO algorithm regarding the effect of increasing the problem size on its performance using Big O notation. The algorithm parameters were set as: population size 25, 500 iterations while the problem size was chosen as [10,100,1000,10,000,100000]. For each value of the problem size, we measured the elapsed time using the FFO algorithm, and we obtained a linear relationship between problem size and time, as shown in Fig. 2. This relationship reveals that the complexity of the FFO algorithm is linear.

## Wilcoxon signed-rank test analysis

The Wilcoxon signed-rank test was conducted to evaluate the statistical significance of the performance differences between the proposed Fertilization Firefly Optimization (FFO) algorithm and several comparison algorithms: ALO, BOA, GWO, PSO, SCA, WOA, DDAO, BA, and TSA. This analysis is based on the results presented in Table 9 on F1 test function and presented in Table 10.

**Table 10:** Wilcoxon signed-rank test p-values comparing FFO algorithm against other optimization algorithms.

Algorithm	P-value
ALO	5.1452761E−10
BOA	5.1452761E−10
GWO	5.1452761E−10
PSO	5.1452761E−10
SCA	5.1452761E−10
WOA	7.7385887E−09
DDAO	5.1452761E−10
BA	5.1452761E−10
TSA	5.1452761E−10

**Fig. 3:**
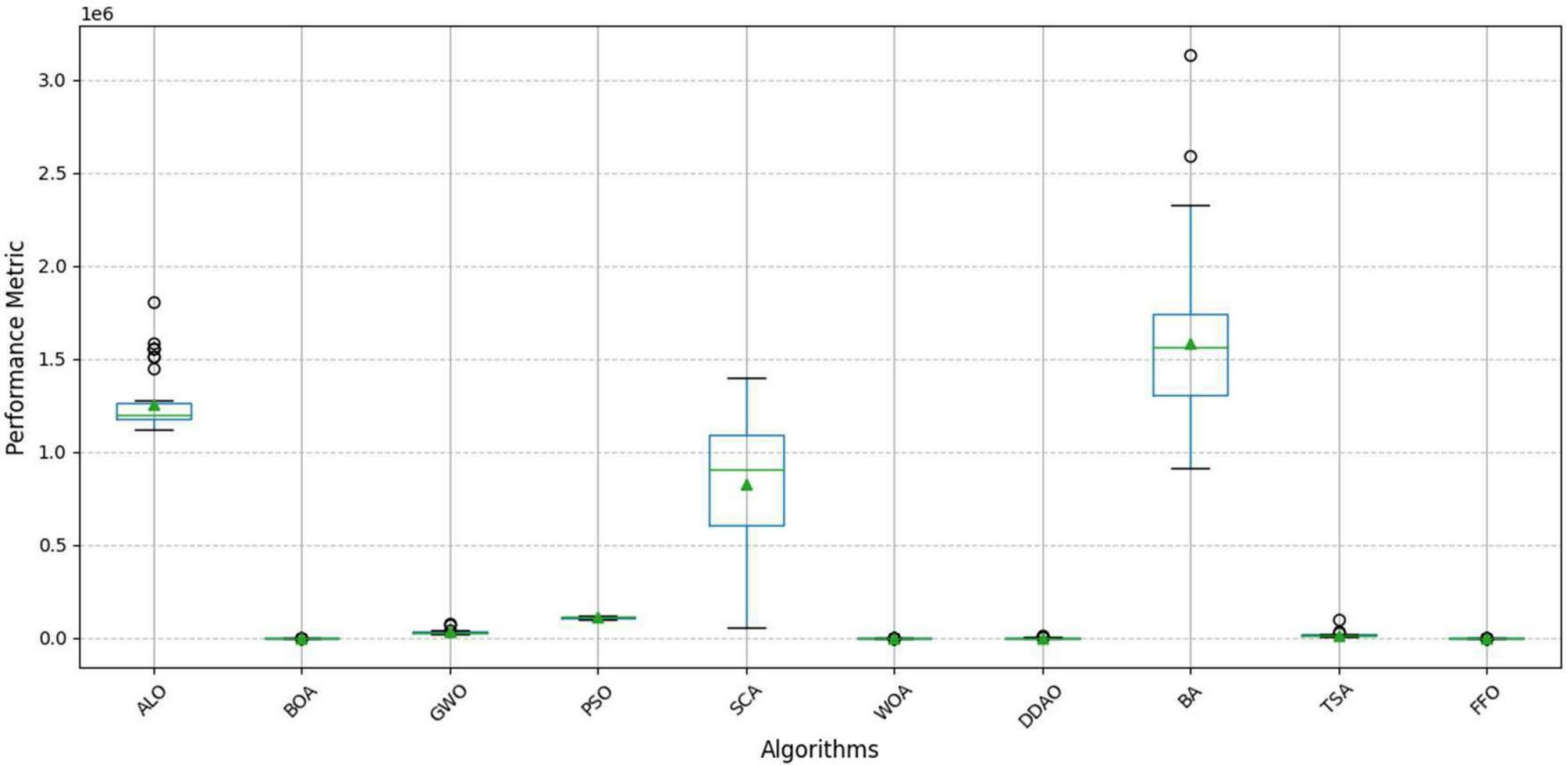
Performance Comparison of Optimization Algorithms Including FFO.

The Wilcoxon test results demonstrate that the FFO algorithm significantly outperforms most comparison algorithms, with p-values well below the 0.05 threshold, providing strong statistical evidence of its superior performance in large-scale optimization problems. The extremely low p-values (e.g., < 0.0001) for algorithms such as ALO, BOA, and GWO strongly support the conclusion that FFO consistently outperforms these methods in optimizing large-scale problems. This reinforces the robustness and efficiency of FFO in solving complex optimization tasks compared to traditional and contemporary algorithms. The test results were derived from 51 independent runs of each algorithm, ensuring statistical reliability and accounting for variations in performance due to random initialization or stochastic behavior. The performance comparison of optimization algorithms is illustrated in Fig. 3, a box plot comparing the distributions of results for all algorithms, revealing the relative performance and variability of the remaining methods. This plot provides a clear understanding of how the algorithms perform. This plot demonstrates FFO’s superior performance and consistency compared to the other algorithms. FFO’s median and interquartile range indicates its robustness and reliability, with fewer outliers and a narrower spread compared to most other methods. The visual distinction further supports the statistical evidence of FFO’s superiority in solving large-scale optimization problems.

**Fig. 4:**
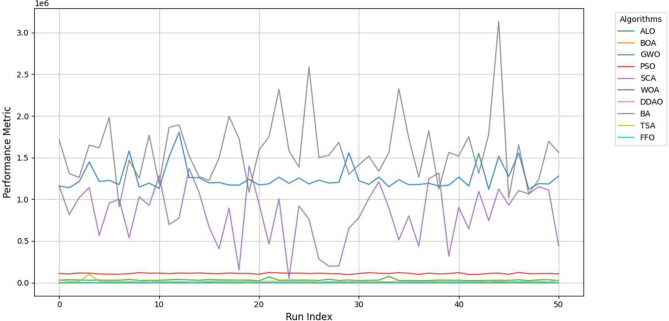
Performance trends of optimization algorithms across 50 independent runs.

Figure 3 illustrates the performance distributions of the optimization algorithms. FFO’s median and interquartile range indicate its robustness, with a narrower spread and fewer outliers than other methods. In contrast, algorithms such as BA and DDAO exhibit wider interquartile ranges, suggesting more significant variability in their performance. The presence of outliers in these algorithms further indicates their inconsistency in solving large-scale optimization problems. To supplement the box plot, Table 10 provides the mean and variance of the performance metrics, offering additional insights into the algorithms’ relative efficiency and reliability. The performance trends, as illustrated in Fig. 4, shows the variability and consistency of optimization algorithms across multiple runs. The trends demonstrate diverse behaviors among the algorithms, with some exhibiting significant variability, indicating sensitivity to initial conditions or problem-specific parameters. In Fig. 4, FFO’s performance is included for direct comparison. The trends reveal that FFO consistently outperforms other algorithms, achieving superior results with minimal fluctuation across all runs. This presents FFO’s robustness and reliability in addressing large-scale optimization problems. FFO’s consistent and stable trends reinforce its superiority, complementing the statistical analysis and validating its effectiveness.

The trends in Fig. 4 illustrate the variability and stability of optimization algorithms across multiple runs. Algorithms such as BA, DDAO, and GWO exhibit significant fluctuations, indicating sensitivity to initialization or difficulty in maintaining convergence in large-scale problems. In contrast, the FFO algorithm consistently achieves superior results with minimal variability.

## Exploitation analysis

The exploitation capability of an optimization algorithm refers to its ability to intensively search and fine-tune the promising areas within the search space to converge toward the global optimum. An algorithm with strong exploitation efficiently hones in on the best solutions by exploring the vicinity of high-quality candidates. In the experiments conducted, the Flower Fertilization Optimization Algorithm (FFO) demonstrated remarkable exploitation abilities, particularly in optimizing unimodal and fixed-dimension benchmark functions.

As shown in Table 3, the FFO algorithm outperformed other metaheuristics on unimodal benchmarks F1–F7. The FFO achieved the lowest best and mean objective function values across all unimodal functions. For instance, on function F1, FFO obtained a best value of 1.570 × 10^−175^ and a mean value of 1.101 × 10^−78^, significantly better than its competitors. The low STD values indicate that FFO consistently finds high-quality solutions across independent runs, showcasing its reliable exploitation capability. The convergence curves in **Error! Reference source not found.** illustrate FFO’s rapid and steady convergence on unimodal functions such as F1, F2, and F3. The FFO exhibits a linear convergence pattern toward the global optimum, reflecting its ability to exploit the local search space efficiently.FFO refines the solutions with each iteration, indicating effective local search and fine-tuning mechanisms.

## Exploration analysis

Exploration in optimization algorithms refers to broadly searching the global search space to discover diverse regions that may contain the global optimum. An effective exploration mechanism helps prevent premature convergence to local optima and ensures a comprehensive solution space search. The FFO algorithm showcased significant exploration capabilities, particularly evident in the results of multimodal and large-scale optimization problems. As depicted in Table 4, FFO achieved superior performance on multimodal benchmarks F8–F16. On functions F9, F10, and F11, FFO consistently found the global optimum across all runs, with best and mean values of zero and a STD of zero, indicating that it effectively explored the search space to locate the optimal solutions. The negligible STD values across these functions suggest that FFO maintains a high level of exploration diversity, allowing it to escape local optima. In Table 5, FFO performed competitively on fixed-dimension benchmarks, which often have complex landscapes with multiple local optima. The FFO obtained solutions that were very close to the global optima, demonstrating its ability to explore the search space effectively despite the functions’ challenging nature. The exploration prowess of FFO is further shown in the large-scale optimization experiments shown in Table 9. Also, FFO excelled in 1000-dimensional functions F29–F32, achieving best values of zero or values extremely close to the global optimum. The FFO outperformed other algorithms, such as Ant Lion Optimizer, Butterfly Optimization Algorithm, and Tunicate Swarm Algorithm, which struggled to maintain performance in high-dimensional spaces. The convergence curves in **Error! Reference source not found.** For multimodal functions such as F10 and F11 show that FFO exhibits an unpredicted convergence manner, with sudden improvements in solution quality, indicative of successful exploration jumps. The algorithm does not get trapped in local optima, as evidenced by its ability to find better solutions in later iterations.

## Sensitivity of the FFO algorithm

In addition to studying the sensitivity of the FFO algorithm to population size in Sect. “Experiments”, the impact of Eq. 5 on the performance of the proposed FFO has been evaluated on two benchmark test functions: the Sphere function and the Rosenbrock function^10^. The results, presented in Table 11, demonstrate a notable sensitivity of FFO to Eq. 5, with the effectiveness of this equation being highly problem-dependent.

**Table 11:** Performance comparison of FFO with and without Eq. 5 on Sphere and Rosenbrock test functions.

Function	Equation 5	
	Not included	Included
Sphere	0.036403	**3.1683E−85**
Rosenbrock	**16.9283**	23.7639

**Fig. 5:**
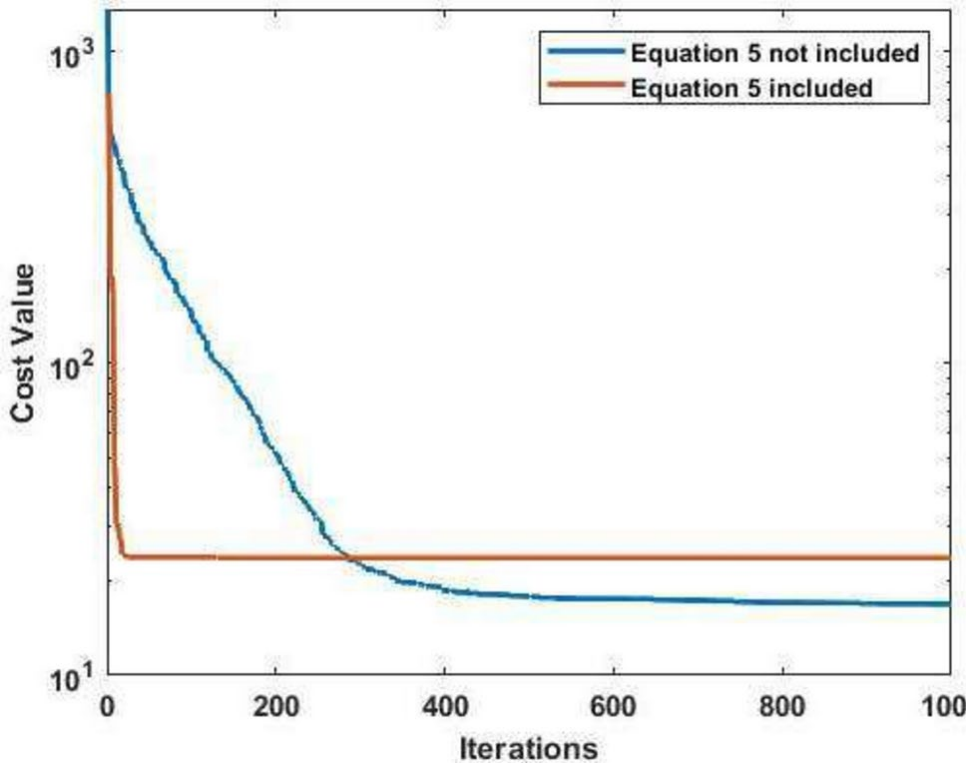
Effect of including Eq. 5 on the convergence curve of the FFO on the Rosenbrock function.

Equation 5 significantly enhances the FFO’s performance, as evidenced by the dramatic reduction in the cost value. Without Eq. 5, the FFO achieves a cost value of 0.036403, whereas the inclusion of Eq. 5 reduces this to an extremely low value of 3.1683E-85. This suggests that Eq. 5 introduces beneficial dynamics that accelerate convergence and improve precision on separable, convex optimization problems like the Sphere function. In contrast, Eq. 5 negatively affects FFO performance for the Rosenbrock function, increasing the cost value from 16.9283 (without Eq. 5) to 23.7639 (with Eq. 5). Equation 5 may disrupt the FFO’s ability to fine-tune solutions in the complex, narrow valleys of non-convex functions like Rosenbrock. Figure 5 provides a visual representation of the disadvantages of Eq. 5 on the FFO performance when applied to the Rosenbrock function. The convergence curve reveals slower progress toward the global minimum and higher stagnation when Eq. 5 is included. This contrasts with the Sphere function, where Eq. 5 clearly accelerates convergence.

The results reveals that the effectiveness of Eq. 5 depends on the nature of the optimization problem. Equation 5 should be applied selectively based on the characteristics of the optimization problem. An adaptive strategy could be developed to dynamically turn Eq. 5 on or off during optimization, depending on intermediate performance metrics. Additional testing on a diverse set of benchmark functions could provide deeper insights into the conditions under which Eq. 5 is beneficial and that can be a matter of future work. Additionally, Table 12 demonstrates that the FFO algorithm exhibits minimal sensitivity to variations in the damping factor $\beta$. This robustness indicates that the proposed FFO algorithm is more straightforward to apply across diverse optimization landscapes, reducing the need for extensive parameter tuning. The results show consistent performance across different $\beta$ values, affirming the adaptability and user-friendliness of the FFO algorithm.

**Table 12:** Sensitivity of the FFO to $\beta$ value.

$\beta$ value	Best	SD
0.99	1.130E−84	2.645E−78
0.95	7.088E−83	2.064E−78
0.8	1.034E−83	3.168E−78
0.7	1.257E−84	6.040E−79

## Optimized PID controller for magnetic train positioning

In the context of control systems, the PID controller is preferred for its simplicity and efficiency in a wide array of industrial applications. Even when advanced control methods have risen, the PID controller is still a dominant tool, especially in processes requiring straightforward implementation and reliability. However, tuning the PID parameters is critical for achieving optimal performance. This task becomes particularly challenging in nonlinear systems such as magnetic train positioning. To address this, advanced optimization techniques are employed to fine-tune PID gains; ${K}_{p},{K}_{i},{K}_{d}$ Ensuring precise control and efficient operation. The methodology for optimizing PID controllers for magnetic train positioning involves several key steps, integrating system modeling, optimization, and validation. The process utilizes the FO algorithm to identify the optimal PID parameters that minimize the control error.

### Dynamics of train movement

The dynamics of train movement along a track are governed by classical mechanics, where the train is modeled as a rigid body subjected to various forces on a frictionless railway. Understanding these dynamics is crucial for designing effective control strategies for precise positioning. Key parameters for modeling the train dynamics are shown in Table 13 with their values and they include the mass of the train ($m$), which affects its inertia and the force required to change its motion. Also, the time step ($dt$), which is the discrete time interval for numerical integration and initial Conditions, the initial position, velocity, and acceleration of the train.

**Table 13:** Parameters of the magnetic train model.

Parameters	Value
$m$ [kg]	100
$\theta$ [deg]	0
$dt$ [sec]	0.02
Train x-position [m]	0
Train y-position [m]	0
object x-position [m]	100
object y-position [m]	6.5

**Fig. 6:**
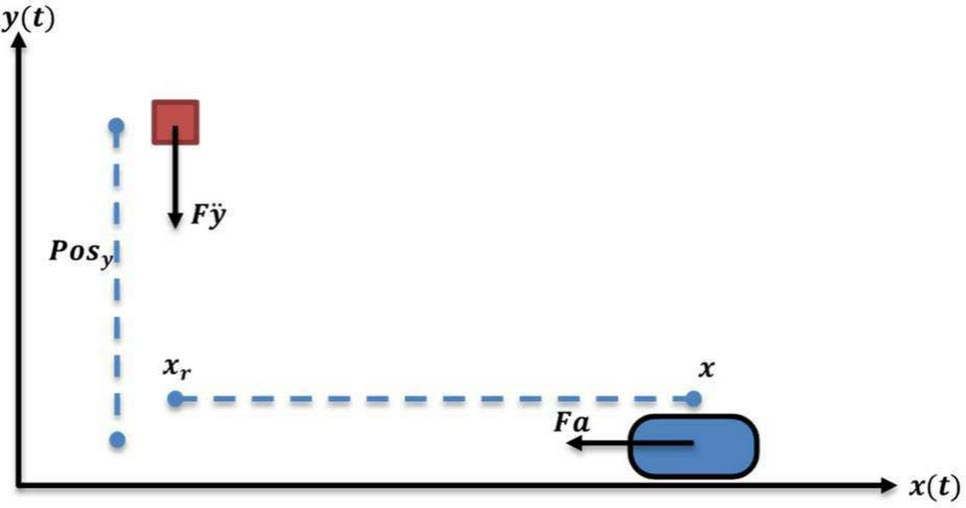
Mathematical model of the magnetic train.

The primary forces on the train are shown in Fig. 6, the control force $Fa$, which is the force applied by the PID controller to adjust the train’s position; this force comes from multiplication of the mass $m$ with linear acceleration $a$.

The motion of the train is described by Newton’s second law, which relates the net force acting on the train to its acceleration. The net force in this example is $Fa$ is the sum of the control force and the other possible forces. Thus, the acceleration of the train can be calculated as

11 $$\ddot{a}=\frac{Fa}{m}$$

Acceleration is the time derivative of the velocity, therefore, Eq. (11) can be rewritten as follows:

12 $$\underset{vi}{\overset{v(t)}{\int }}dv=\frac{1}{m}\underset{to}{\overset{t}{\int }}Fa dt$$

where ${t}_{O}$ and $t$ are the initial time and time interval, respectively and $vi$ is the initial velocity. The velocity $v$ and displacement $x$ of the train are updated using numerical integration.

13 $$\underset{xi}{\overset{x(t)}{\int }}dx=\underset{to}{\overset{t}{\int }}v dt$$

14 $$x\left(t\right)={x}_{i}+\underset{to}{\overset{t}{\int }}v dt$$

The dynamics of the magnetic train are modeled using Newton’s second law, with the PID controller providing the control force. By combining the equations for the train’s dynamics and the PID controller, the transfer function of the system is derived as follows:

15 $$G\left(s\right)=\frac{X(s)}{R(s)}=\frac{{K}_{p}.{s}^{2}+{K}_{d}. {s}^{3}+{K}_{i}. s}{m. {s}^{3}}$$

where $G\left(s\right)$ is the transfer function of the system. ${K}_{p}$, ${K}_{d}$, and ${K}_{i}$ are proportional, integral, and derivative gains of the PID controller. *m* is the mass of the train, $X(s)$ is Laplace transform of the train’s position, and $R(s)$ is Laplace transform of the reference position.

### Control challenges in train positioning

**Fig. 7:**
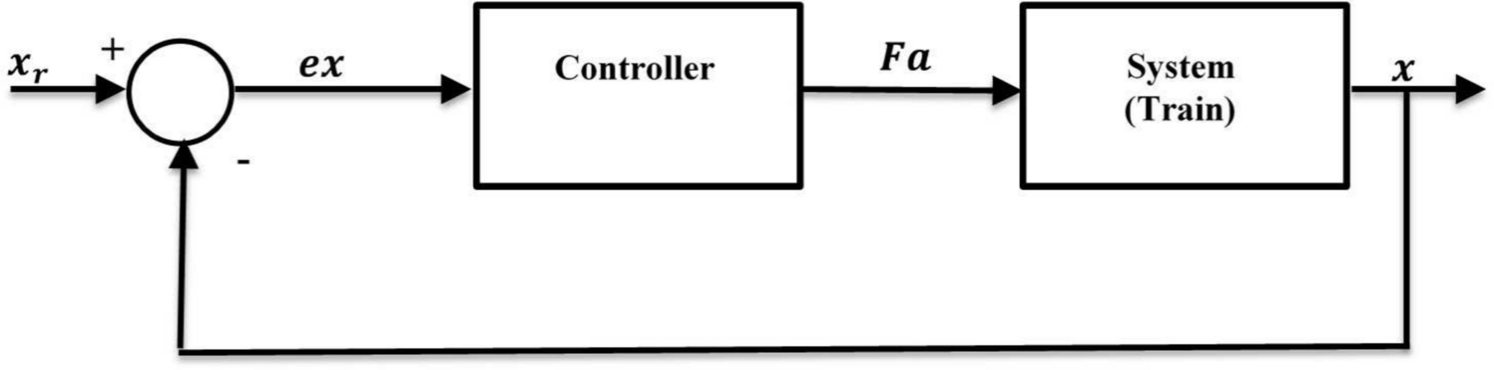
Control system of a magnetic train.

Figure 7 reveals the control system of the magnetic train where the input to the controller is the error in position calculated as

16 $$ex={x}_{r}-x,$$

while the output of the controller is the control force, which is the input to the train system. When the train is far away from the target, a large control force should be applied to the plant; otherwise, the train will not reach in time to catch the object. When the train is close to the target, a small amount of control force should be applied; otherwise, the train will overshoot and fail to catch the target.

Ensuring precise control of train positioning is a complex task that involves addressing several challenges due to the dynamic nature of the railway environment. The dynamics of train movement are inherently nonlinear due to varying track conditions, changing loads, and external disturbances. Nonlinearities can arise from factors such as friction, air resistance, and the train’s interaction with the track. Nonlinear behavior complicates the design of control systems because linear control techniques may not perform adequately.

### PID control for train positioning

**Fig. 8:**
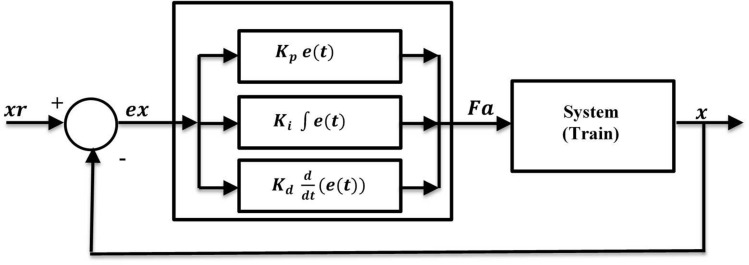
PID Controller of the magnetic train.

Proportional-Integral-Derivative (PID) controllers are widely used due to their simplicity and effectiveness. They adjust the control input based on the error, accumulated error, and rate of change of error to maintain the desired position. In PID controller, the control force is calculated based on the error, which is the difference between the reference and actual positions, as shown in Fig. 8.

17 $$Fa={K}_{p} e\left(t\right)+{K}_{i} \int e(t)+{K}_{d} \frac{d(e(t))}{dt}$$

where ${K}_{p}$, ${K}_{i}$, and ${K}_{d}$ are coefficients of the PID controller that have to be chosen precisely. The PID controller is a simple and efficient technique to control systems, yet the challenge is how to tune its coefficients for optimal performance.

### Optimized-ANN controller

In this section, we describe the multi-objective optimization problem used to tune the PID controller parameters proportional gain (${K}_{p}$), integral gain (${K}_{i}$), and derivative gain (${K}_{d}$) for the magnetic train system. The FFO algorithm was employed to minimize multiple objectives simultaneously. The objectives were chosen to balance the performance criteria of the PID controller, ensuring a robust and efficient control system. The optimization problem is formulated as a multi-objective problem with the following objectives:

1. Minimization of Overshoot ${O}_{s}$

18 $${O}_{s}=\text{max}\left(displacement \left(t\right)\right)-ref position$$

This objective ensures that the system’s response does not exceed the desired reference position excessively, which could lead to instability or damage.

2. Minimization of Settling Time ${T}_{s}$

19 $${T}_{s}=min\left\{t \Vert displacement \left(t\right)-ref position\Vert <\epsilon , \forall t \ge {T}_{s}\right\}$$

This equation ensures that the system’s response reaches and stays within a specified tolerance $\epsilon$ of the reference position as quickly as possible. Minimizing this time leads to faster system stabilization.

3. Minimization of Steady-State Error ${e}_{ss}$

20 $${e}_{ss}=\underset{t\to \infty }{\text{lim}}\left|displacement \left(t\right)-ref position\right|$$

Steady-state error measures the final difference between the system’s position and the reference position. Minimizing this ensures that the system accurately tracks the desired reference.

4. Minimization of Control Effort ${E}_{u}$

21 $${E}_{u}=\underset{0}{\overset{t}{\int }}{E}_{u} dt$$

where ${u}_{t}$ is the control input (force applied to the train). Minimizing control effort reduces energy consumption and wear on the system components. The optimization process is subject to the following constraints:

$$100\le {K}_{p}\le 500$$

$$10\le {K}_{i}\le 300$$

22 $$100\le {K}_{d}\le 500$$

These bounds ensure that the PID gains remain within a practical and stable range. Thus the overall fitness function can be written as:

23 $$f={W}_{1} {O}_{s}+{W}_{2} {T}_{s}+{W}_{3} {e}_{ss}+{W}_{4} {E}_{u}$$

where ${W}_{1}$, ${W}_{2}$, ${W}_{3}$, and ${W}_{4}$ are weights. The FO was employed to solve the multi-objective optimization problem by iteratively improving the PID gain values to minimize the objectives. The optimization process was executed multiple times with random initial conditions for the train and passenger positions. The resulting optimal PID gain values and their corresponding performance metrics (overshoot, settling time, steady-state error, and control effort) were collected to form a comprehensive dataset. This dataset was then used to train an Artificial Neural Network model^70^ to predict optimal real-time PID gains for new initial conditions. In other words, a set of reference points representing different possible train positions are handled to the FFO algorithm to return the optimal PID gains. The set of reference points and their corresponding PID gains form a dataset that can be used to train an ANN. The resulting trained ANN will be able to predict the best gains for a given reference point, resulting in a sophisticated adaptive controller. The weight factors in Eq. (23) were determined based on the relative importance of the objectives. Higher weights were assigned to overshoot, and steady-state error was assigned to prioritize accuracy and reliability while settling time and control effort were weighted to ensure efficient system operation. These weights represent a balance that supports stable and robust system behavior. Adjusting these weights would shift the emphasis among objectives, potentially impacting system stability and performance trade-offs.

### PID optimization statistical results

The FFO algorithm was benchmarked against PSO, DDAO, and AEABC in a multi-objective PID optimization problem. The experiment involved 100 random configurations of magnetic train and passenger positions to evaluate each algorithm’s performance comprehensively. The results in Table 14 summarize the sum of mean errors and standard deviation for each algorithm.

**Table 14:** FFO algorithm and other metaheuristics on Multi-objective PID parameters optimization.

Algorithm	Sum of mean errors	SD
FFO	190.563	8.687
PSO	250.075	7.861
DDAO	219.629	9.872
AEABC	22,922.585	1541.16

The results indicate that the FFO algorithm outperformed the other optimization methods, achieving the lowest sum of mean errors and maintaining a competitive standard deviation. This demonstrates FFO’s ability to fine-tune PID parameters, leading to more precise and stable control system performance. While AEABC exhibited the highest standard deviation and error sum, FFO consistently provided reliable results across the tested configurations, illustrating its robustness and efficiency in handling nonlinear control challenges. These findings validate FFO’s effectiveness as a metaheuristic optimization tool, particularly for complex multi-objective problems in engineering applications.

### System setup

This study employed an artificial neural network ANN to predict the optimal PID gains for controlling a magnetic train system. The ANN^71^ used was a multi-layer perceptron with two hidden layers, each consisting of 100 neurons. This architecture was chosen due to its ability to approximate complex nonlinear functions, making it suitable for mapping the relationship between the initial conditions of the train and the reference positions to the optimal PID gains. The dataset used to train the ANN was generated through the FFO-based multi-objective optimization process, which produced 100 observations. Each observation in the dataset included the initial train position, reference position, and the corresponding optimal PID gains obtained from the optimization process. The training process involved splitting the dataset into training and testing sets, where 80% of the data was used for training and 20% for testing. Standard scaling was applied to both the input features and the target outputs to normalize the data, enhancing the training stability and performance of the ANN. The training of the ANN was performed using the backpropagation algorithm, with a mean squared error loss function and an adaptive learning rate. The model converged well, achieving a satisfactory R^2^ score on the test set, indicating that the ANN effectively captured the underlying patterns in the data. The trained ANN demonstrated the ability to generalize to new scenarios, providing accurate predictions of PID gains for various initial conditions, as evidenced by the low steady-state error and rapid settling times observed in the simulations. This performance indicates the potential of using ANNs for real-time adaptive control in dynamic systems like the magnetic train.

### Simulation

**Fig. 9:**
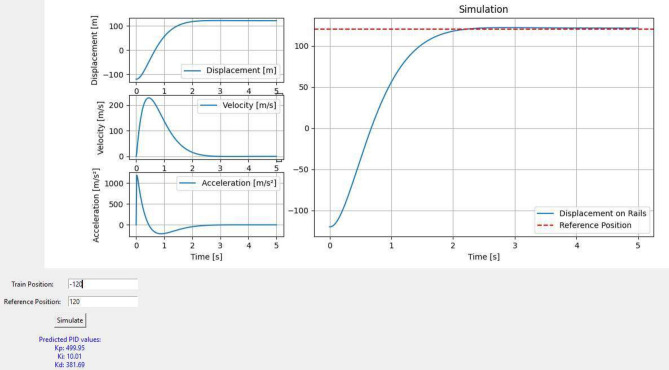
Optimized-ANN PID controller simulator.

An interactive simulation was developed to visualize the controller’s performance in real-time and illustrate the effectiveness of the ANN-based PID controller. In this example, the simulator is set up to control a magnetic train, where the objective is to move the train from its initial position to a reference position on a rail. The train’s and reference positions can be adjusted through a user interface, allowing various scenarios to be tested. In the illustrative example shown in Fig. 9, the train is initially positioned at (− 120) m, while the reference position is set at (120) m. The ANN-based PID controller predicts the optimal gains based on these positions, which are then used to control the train’s movement along the rail. The ANN model used in this simulation was trained using a dataset of 100 observations generated from an optimization process. The model was designed to predict three PID gains based on the initial and reference positions. For this specific scenario, the ANN model predicted:

$${K}_{p}=499.95$$

$${K}_{i}=10.01$$

$${K}_{d}=381.69$$

These gains were then applied to the control system, and the resulting behavior of the train was visualized in the simulator. The results of the simulation are depicted in Fig. 9. The main plot on the right shows the displacement of the train over time, with the reference position marked as a red dashed line. The train successfully moves towards the reference position and stabilizes near it, demonstrating the effectiveness of the ANN-based PID controller.

The plots on the left side in Fig. 9 provide additional insights into the system’s dynamics:

- This plot confirms that the train’s position converges smoothly towards the reference point, with minimal overshoot and settling time.
- The train’s velocity increases initially as it accelerates towards the reference position, then decreases as it approaches and stabilizes near the reference point.
- The acceleration plot showing the force applied to the train decreases as the train approaches the reference position, indicating that the controller effectively manages the system’s dynamics.

This application demonstrates the efficiency of the FFO algorithm and its applicability to engineering control problems.

## Conclusion

Our research introduces the Flower Fertilization Optimization Algorithm (FFO), a new metaheuristic optimization method inspired by how flowering plants naturally fertilize. In the same way pollen grains travel to find and fertilize ovules, the FFO blends exploring many solutions with zooming in on the best ones. To test the FFO, we ran it on 32 different optimization problems—covering everything from simple unimodal tasks to trickier multimodal and fixed-dimension ones. We compared FFO against 14 top-tier algorithms, including Particle Swarm Optimization, Grey Wolf Optimizer, Ant Lion Optimizer, Harris Hawks Optimizer, and Butterfly Optimization Algorithm. Across most tests, FFO outperformed the others by consistently delivering better mean and standard deviation values, reflecting its accuracy and reliability. On unimodal functions, FFO showed a knack for quickly homing in on the global optimum, underscoring its strength in precise fine-tuning. For more complex and larger-scale tasks, FFO avoided getting stuck too soon and kept a healthy variety of solutions in the mix, which shows it does a great job of exploring. It also scaled well to problems with up to 100,000 variables, confirming its linear time complexity and its suitability for real-world tasks with high dimensionality. Beyond artificial benchmarks, we put the FFO to work on a real challenge: tuning a PID controller for magnetic train positioning. Because setting PID parameters correctly is key to good performance, we used the FFO to minimize control errors and boost stability. In our tests, the FFO-optimized controller beat traditional methods, delivering more accurate positioning and faster response times while maintaining stability under different conditions. This real-world success highlights how FFO can shine in engineering problems that need precise and reliable optimization. As a powerful and flexible tool, FFO shows strong promise for contributing to many scientific and engineering fields, promoting both innovation and efficiency. However, like other metaheuristics, FFO does have its constraints. It works within a chosen search space, and picking that space can be hard for real-world issues. Tuning parameters like the velocity reduction coefficient and damping factor also needs careful attention for the best results. Certain strategies—for instance, ones like Eq. 5—may boost FFO’s performance in some areas but hurt it in others, pointing to a need for adaptive methods. And while FFO’s runtime grows only linearly with larger problem sizes, that can still be a concern for extremely high-dimensional cases. Despite these challenges, FFO holds a valuable spot in the optimization research community. After seeing how well it worked for PID tuning, we believe it could succeed in other areas like robotics, supply chain optimization, and renewable energy. Future work may focus on adding adaptive parameter tuning or combining FFO with other optimization techniques to make it even more effective.

## Data Availability

The dataset generated and analyzed during this study is available at: https://www.kaggle.com/datasets/hazimbedran/magnetic-train-on-a-straight-railway.
